# Retrieval Practice Enhances New Learning but does Not Affect Performance in Subsequent Arithmetic Tasks

**DOI:** 10.5334/joc.216

**Published:** 2022-03-22

**Authors:** Bernhard Pastötter, Julian Urban, Johannes Lötzer, Christian Frings

**Affiliations:** 1 Department of Psychology, University of Trier, DE

**Keywords:** Memory, Learning, Long-term memory, Working memory

## Abstract

The forward testing effect is an indirect benefit of retrieval practice. It refers to the finding that retrieval practice of previously studied information enhances learning and retention of subsequently studied other information in episodic memory tasks. Here, two experiments were conducted that investigated whether retrieval practice influences participants’ performance in other tasks, i.e., arithmetic tasks. Participants studied three lists of words in anticipation of a final recall test. In the testing condition, participants were immediately tested on lists 1 and 2 after study of each list, whereas in the restudy condition, they restudied lists 1 and 2 after initial study. Before and after study of list 3, participants did an arithmetic task. Finally, participants were tested on list 3, list 2, and list 1. Different arithmetic tasks were used in the two experiments. Participants did a modular arithmetic task in Experiment 1a and a single-digit multiplication task in Experiment 1b. The results of both experiments showed a forward testing effect with interim testing of lists 1 and 2 enhancing list 3 recall in the list 3 recall test, but no effects of recall testing of lists 1 and 2 for participants’ performance in the arithmetic tasks. The findings are discussed with respect to cognitive load theory and current theories of the forward testing effect.

## Introduction

It is a prominent finding that retrieval practice enhances long-term retention of previously studied material more than other forms of reprocessing the material (e.g., restudy or study with concept mapping; Karpicke & Blunt, 2011; Roediger & Karpicke, 2006). This *direct* benefit of retrieval practice can be referred to as backward testing effect (Pastötter & Bäuml, 2014; for reviews, see Karpicke, 2017; Roediger & Butler, 2011). In addition, there are *indirect* benefits of retrieval practice for long-term episodic memory and learning. For instance, retrieval practice can potentiate relearning of previously studied and practiced material, an effect that has been referred to as test-potentiated learning (e.g., Arnold & McDermott, 2013). Even more striking, retrieval practice can enhance subsequent *new* learning of previously not studied and not practiced material, which has been referred to as forward testing effect or test-potentiated new learning (e.g., Pastötter, Schicker, Niedernhuber, & Bäuml, 2011; Szpunar, McDermott, & Roediger, 2008). While there has been extensive research on the benefits of retrieval practice for participants’ performance in episodic memory tasks, the present study was designed to investigate whether retrieval practice influences participants’ performance in other tasks, i.e., arithmetic tasks (modular arithmetic and a single-digit multiplication), which follow retrieval practice.

### Forward Testing Effect

The forward testing effect is typically examined in a multi-list learning environment, in which participants study several (e.g., three) lists of items in anticipation of a final recall test. In the testing condition, participants are immediately tested on lists 1 and 2 after study of each list, whereas in the restudy condition, they restudy lists 1 and 2 after initial study. Next, all participants study and are tested on list 3, which is the critical list. Finally, lists 1 and 2 are tested in final tests. The typical finding is that interim testing of lists 1 and 2 enhances correct recall of list 3 and reduces the number of prior-list intrusions in the list 3 recall test (for reviews, see Pastötter & Bäuml, 2014; Yang, Potts, & Shanks, 2018). The forward testing effect is a robust effect that is observed for different materials (e.g., words, texts, videos; e.g., Bäuml & Kliegl, 2013; Szpunar, Khan, & Schacter, 2013; Wissman, Rawson, & Pyc, 2011; Yang, Chew, Sun, & Shanks, 2019) and is broadly present in different populations (e.g., children, older adults, patients; e.g., Dang, Yang, & Chen, in press; Pastötter & Bäuml, 2019; Pastötter, Weber, & Bäuml, 2013). In addition, the forward testing effect shows significant retest-reliability (Pastötter & Frings, 2019), is independent of learners’ working memory capacity (Pastötter & Frings, 2019; Yang et al., 2020), and is immune to acute psychosocial stress (Pastötter, von Dawans, Domes, & Frings, 2020).^1^

The forward testing effect is a multi-mechanism phenomenon (Kliegl & Bäuml, 2021; Yang et al., in press). According to Yang et al. (in press), three prominent theories of the forward testing effect are release-from-proactive-interference (PI), reset-of-encoding, and strategy-change. The release-from-PI theory assumes that testing promotes context change, i.e., contextual segregation of the item lists, which reduces proactive interference between lists at test and thus enhances recall of the critical list (Bäuml & Kliegl, 2013; Szpunar et al., 2008). The reset-of-encoding theory also assumes that interim testing promotes context change; in addition, however, this theory postulates that the segregation “resets” the encoding process and thus reduces memory load and inattention during the encoding of subsequently studied information (Pastötter, Engel, & Frings, 2018; Pastötter et al., 2011). The strategy-change theory suggests that testing induces participants to switch to more elaborative encoding and/or more effective retrieval strategies for further learning (Chan, Manley, Davis, & Szpunar, 2018; Cho, Neely, Crocco, & Vitrano, 2017). In addition, metacognitive and integration theories have been suggested that explain the forward testing effect by enhanced motivation toward attentional encoding and/or effortful retrieval, increased test expectancy, and increased integration of the tested and newly studied item material (Cho et al., 2017; Weinstein, Gilmore, Szpunar, & McDermott, 2014; Wissman et al., 2011; for a review, see Chan, Meissner et al., 2018).

Recent research has only just begun to investigate which mechanism(s) exactly contribute(s) to the forward testing effect under which experimental conditions and factors. For example, Kliegl and Bäuml (2021) recently examined the influence of the duration of the retention interval between critical list learning and final recall testing on the forward testing effect for categorized versus unrelated word lists. The results showed that the forward testing effect for the categorized item material was equally present for both the short (1 min) and the relatively long (25 min) retention interval, whereas the forward effect for the unrelated material was present for the short but absent for the long retention interval. Based on these results, Kliegl and Bäuml (2021) suggested a two-factor explanation, according to which the forward testing effect is mainly driven by context change (i.e., release-from-PI and reset-of-encoding) with unrelated item material and is mainly driven by strategy change with categorized item material. The present study used unrelated word lists as item material. Thus, according to the two-factor account, release-from-PI and reset-of-encoding can be considered the main factors for the forward testing effect in the present memory task. The predictions of the release-from-PI and reset-of-encoding theories for the impact of retrieval practice on participants’ performance in the arithmetic (working memory) tasks are presented below.

### Cognitive Load Theory

The present study was designed to examine whether retrieval practice influences participants’ performance in subsequent arithmetic tasks, i.e., the modular arithmetic task and single-digit multiplication task. In these tasks, performance has been suggested to be based to a certain degree on the available working memory resources of the participants. In the literature, the concept of cognitive resources in general and working memory resources in particular is an integral part of information processing theories. One such theory is the cognitive load theory (Sweller, Ayres, & Kalyuga, 2011). It assumes that long-term memory is virtually unlimited in capacity, whereas working memory is limited in capacity and also in duration over which it can hold information. Furthermore, cognitive load theory assumes that working memory load can be reduced if the information that is processed is partially stored in long-term memory. Accordingly, working memory load is relatively high when participants do a hitherto unknown arithmetic task, e.g., modular arithmetic problems like “25 = 4 (mod 7)” (e.g., Beilock & Carr, 2005; Runge, Frings, & Tempel, 2019; see Methods of Experiment 1a), but relatively low when participants solve single-digit multiplication problems, e.g., “6 × 6 = ?”, for which the solution is stored in long-term memory. Cognitive load theory additionally assumes that individual working memory capacity is not fixed but can be depleted after cognitively demanding tasks. For instance, Schmeichel (2007) showed that inhibiting predominant writing tendencies in a story writing task decreased subsequent working memory capacity, whereas Runge et al. (2019) demonstrated that memory offloading due to saving previously studied information on a computer can preserve working memory resources. In addition, it has been suggested that spacing of learning (Chen, Castro-Alonso, Paas, & Sweller, 2018) and retrieval practice (Leahy & Sweller, 2019) can deplete working memory resources in children. Indeed, it is a prominent idea in the testing effect literature that retrieval practice requires more mental effort than restudy does, which leads to more elaborative processing and thus better long-term retention after testing (Bjork & Bjork, 1992; Pyc & Rawson, 2009; for corresponding evidence, see Coppens, de Jonge, van Gog, & Kester, 2020; Endres & Renkl, 2015; who examined the effects of retrieval practice with different test formats in comparison to restudy on participants’ perceived mental effort).

Leahy and Sweller (2019) examined the backward testing effect in 9-to-10-year old children. Two experiments were conducted, in which the children did a rule learning task. The materials were either repeatedly studied (study-only) or both studied and retrieval practiced (study-test). In one experiment, the children were explained how to construct persuasive arguments; in the other experiment, they were instructed how to create puzzle poems. Individual working memory capacity was measured with a reading span task (Daneman & Carpenter, 1980) for children. The working memory test was conducted either immediately or seven days after the rule learning task. Finally, children’s knowledge about how to construct persuasive arguments or how to create puzzle poems was assessed in a final criterion test that followed the working memory test. In this final test, a backward testing arose after long but not after short delay (see also Roediger & Karpicke, 2006; Toppino & Cohen, 2009). However, the results of the working memory test were less clear-cut. In the first experiment, retrieval practice did not affect working memory performance, neither after short nor after long delay. In the second experiment, retrieval practice impaired working memory performance significantly after short delay but not significantly after long delay. However, the interaction between study/practice conditions and delay was not significant. Leahy and Sweller (2019) interpreted these results in favor of the cognitive load theory, according to which retrieval practice is more demanding than repeated study and therefore working memory resources were depleted after short delay and recovered after long delay. However, we disagree with this conclusion because comparisons between two effects should not be interpreted when the interaction is not significant (see Makin & Orban de Xivry, 2019). We therefore argue that new experiments with higher statistical power are needed to re-examine this issue. The present experiments were designed to take a first step in this direction.

### The Present Study

Two experiments are reported that examined whether retrieval practice influences adult participants’ performance in subsequent arithmetic tasks. Two tasks were selected that rely on working memory resources to a greater (modular arithmetic task; Experiment 1a) or a lesser degree (single-digit multiplication task; Experiment 1b). In both experiments, participants went through a testing and a restudy condition. In each condition, participants studied three lists of words, which they were asked to remember for a final recall test. In the testing condition, participants were tested on lists 1 and 2 after studying each single list, whereas in the restudy condition, they restudied lists 1 and 2 after initial study. In both conditions, participants completed an arithmetic task in a first block before list 3 learning and in a second block after list 3 learning. The tasks were a modular arithmetic task in Experiment 1a and a single-digit multiplication task in Experiment 1b. Notably, both tasks have been shown to be sensitive to experimental manipulations in adults in previous studies (e.g., Campbell & Thompson, 2012; Runge et al., 2019). Finally, participants were asked to recall list 3, list 2, and list 1. The critical dependent variables were correct recall and the number of prior-list intrusions in the list 3 criterion test of the episodic-memory task and the number of correct answers in each of the arithmetic tasks. Regarding participants’ performance in the list 3 criterion test, we expected to observe a forward testing effect, i.e., enhanced correct recall of list 3 items and reduced number of prior-list intrusions in the testing condition than the restudy condition (e.g., Pastötter et al., 2018; Pastötter & Frings, 2019). Data collections of the two experiments were carried out during the same period of time and the participants were randomly assigned to the two experiments. Therefore, the methods and results of the two experiments are reported together.

Regarding participants’ performance in the arithmetic tasks, the release-from-PI and reset-of-encoding theories of the forward testing and the cognitive load theory make different predictions. The release-from-PI theory predicts no effect of testing versus restudy on participants’ performance in the arithmetic tasks. Indeed, build-up (in the restudy condition) and release from PI (in the testing condition) should be specific to the memory task and switching to a substantially different task (i.e., the modular arithmetic task in Experiment 1a and the single-digit multiplication task in Experiment 1b) should not result in any interference effect from preceding list learning and retrieval practice activities in this task. The reset-of-encoding theory assumes that testing reduces subsequent memory load and inattention and thus predicts a potentially positive effect of testing on participants’ performance in the arithmetic tasks. Specifically, a positive effect should be observed in the first block of the arithmetic tasks before list 3 learning, but not in the second block after list 3 learning. Such result would be consistent with earlier serial position findings in the memory task, which showed a reset-of-encoding effect for the primacy items of the target list (Dang et al., in press; Pastötter et al., 2018). Finally, cognitive load theory predicts a negative effect of testing compared to restudy. Indeed, if retrieval practice is cognitively more demanding than restudy, working memory resources should be depleted and participants’ performance in the arithmetic tasks should be impaired after testing compared to restudy. More specifically, because participants’ working memory load should be relatively higher in the more demanding modular arithmetic task (Beilock & Carr, 2005; Runge et al., 2019) than in the single-digit multiplication task, which more strongly relies on long-term memory retrieval (LeFevre et al., 1996), cognitive load theory predicts a relatively larger negative effect of testing in Experiment 1a compared to Experiment 1b.

## Method

### Participants

Sixty-four undergraduate students from Trier University (mean age: 21.64 years, SD = 3.29 years; 53 females, 11 males) participated in Experiment 1a and another 64 undergraduate students (mean age: 21.58 years, SD = 4.16 years; 54 females, 10 males) participated in Experiment 1b. The required sample size for each experiment was calculated with G*Power (v3.1.9.4; Faul, Erdfelder, Buchner, & Lang, 2007). Given α = 0.05 and desired power of 1 – β = 0.95 to detect an effect of testing versus restudy on participants’ performance in the modular arithmetic task with medium effect size, *d* = 0.50, a minimal sample size of 54 participants was calculated. All participants gave written informed consent before participation and received course credit in return for participation. The study was carried out in accordance with the recommendations of the Declaration of Helsinki and approved by the local ethical review committee at the University of Trier.

### Material

For the list learning task, which was identical in both experiments, the material was taken from Pastötter, Kliegl, and Bäuml (2012; Exp. 2). The material consisted of 144 unrelated German nouns of medium frequency and word length between 4 and 8 letters; the words were drawn from CELEX database (Duyck, Desmet, Verbeke, & Brysbaert, 2004). For each participant, 72 (out of the 144) words were randomly drawn and assigned to six 12-item lists. Three of these lists were used in the testing condition, the other three in the restudy condition.

For the arithmetic task in Experiment 1a, the material was taken from Runge et al. (2019). Each participant solved 27 modular arithmetic problems throughout the experiment: 3 problems in the training phase, 12 problems in the restudy condition (6 before and 6 after study of list 3), and 12 in the testing condition (6 before and 6 after study of list 3). Each problem consisted of the term “X = Y (mod Z)”. Participants were instructed to subtract Y from X and divide the subtraction result by Z. They had to decide whether the division result was an integral number or not. For half of the problems, the correct division result was an integral number, e.g., for “33 = 17 (mod 4)” the correct result is “4”, whereas for the other half it was not, e.g., for “49 = 13 (mod 7)” the correct result is “5.14”.

For the arithmetic task in Experiment 1b, single-digit multiplication problems were chosen. Each problem consisted of the term “X * Y = __”, with X and Y being quasi-randomly assigned numbers from 3 to 9, e.g., “6 * 7 = __”. Care was taken to ensure equal distribution of the numbers from 3 to 9 over the single multiplication problems. Same digit products, e.g., “7 * 7 = __”, were included.

### Procedure

#### Experiment 1a

**Figure 1:**
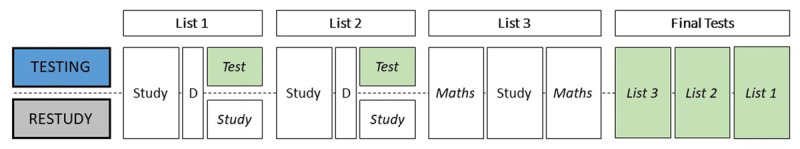
Procedure. Participants studied three item lists, each consisting of 12 words. Study of lists 1 and 2 was followed by a symmetry judgment task as a short distractor (D). In the testing condition, participants were tested on lists 1 and 2 after initial study, whereas in the restudy condition, they restudied lists 1 and 2 after initial study. In both conditions, both before and after study of list 3, participants did an arithmetic task (Exp. 1a: modular arithmetic problems, Exp. 1b: single-digit multiplication problems). Finally, list 3 was tested first, list 2 second, and list 1 last.

Participants took part in both the testing and the restudy condition, with order of conditions counterbalanced across participants. In both conditions, participants studied three 12-item lists (see ***Figure 1***). The items of the three lists were visually presented in random order in the middle of a computer screen with an item presentation rate of 3.75 sec (3 sec item presentation, 0.75 sec blank screen; 45 sec overall). All words were shown in white font color on black background. Subsequent to each presentation of lists 1 and 2, participants did a 30 sec symmetry judgment task as a distractor, in which they were asked to judge whether checkered shapes were symmetrical along the middle vertical axis or not (material taken from Foster et al., 2015). Each distractor consisted of 10 new shapes that were shown with a presentation time of 3 sec. Experimental conditions differed in inter-list activity that followed the symmetry judgment task after lists 1 and 2: In the testing condition, participants were given 45 sec to recall in any order they wished as many items as they could from the just-studied list; in the restudy condition, participants were re-presented the items of the just-studied list in new random order (45 sec presentation time for each list). In both conditions, both before and after study of list 3, participants solved six new modular arithmetic problems, each presented for 7 sec and followed by a 0.5 sec blank screen. Here, the procedure and data analysis closely followed the procedure and analysis used in the study by Runge et al. (2019). Responses were counted as correct if participants typed the correct answer (the division result was an integral number or not) on a (QWERTZ) computer keyboard within the fixed 7 sec presentation time. No feedback was provided. After this, participants were given 45 sec to recall in any order they wished as many items they could from list 3. The list 3 recall test was followed by list 2 and list 1 recall tests (45 sec each). List 1 was always tested last. In all recall tests, participants typed in responses on the computer keyboard. Between conditions, i.e., after the first half of the experiment, participants did a Sudoku for 3 min. A short training phase was included at the beginning of the experiment, in which participants were shown 2 checkered shapes of the symmetry judgment task and 3 modular arithmetic problems.

#### Experiment 1b

The procedure of Experiment 1b was identical to the procedure of Experiment 1a, with the two exceptions that (i) a different arithmetic task, i.e., single-digit multiplication problems, was used and (ii) participants were not trained on this task. In both the testing and the restudy condition, the multiplication problems were shown in the middle of the screen in one block before and one block after study of list 3. Each block lasted 45 sec. Participants were asked to type in correct responses on the computer keyboard and confirm responses by pressing the enter button. They were instructed to solve as many problems as they could during the 45 sec intervals. Immediately after participants pressed the enter button, the next multiplication problem was shown. No feedback was provided. Both experiments were run with E-Prime software (v2.0; Psychology Software Tools, Pittsburgh, PA; see program files on OSF; *https://osf.io/v4cqu/*).

### Data Analysis

Regarding the list learning task, both recall rates of correctly recalled items belonging to a list and prior-list intrusions were examined. First, list 3 recall rate was analyzed as a function of experimental condition in a one-tailed paired samples *t* test (alternative hypothesis: testing > restudy). Second, the number of prior-list intrusions in the list 3 recall test was also examined as a function of experimental condition in a one-tailed paired samples *t* test (alternative hypothesis: testing < restudy); list 1 and list 2 items that were falsely recalled by participants in the list 3 recall test were considered as intrusions. Third, regarding immediate recall of lists 1 to 3 in the testing condition, list recall rate was examined as a function of list (list 1, list 2, list 3) in a repeated measures analysis of variance (ANOVA); Greenhouse-Geisser (GG) correction was applied where necessary. Fourth, regarding final recall of lists 1 and 2, list recall rate was examined as a function of condition (testing, restudy) in two separate two-tailed paired samples *t* tests (alternative hypotheses: testing ≠ restudy).

Regarding the modular arithmetic task in Experiment 1a, the number of correct answers within blocks was examined as a function of experimental condition (testing, restudy) and block (before study of list 3, after study of list 3) in a repeated measures ANOVA. Both errors and omissions were counted as incorrect answers. Mean reaction time for all correct answers was 4.64 sec across blocks and conditions. Regarding participants’ performance in the multiplication task of Experiment 1b, number of correctly solved multiplication problems within blocks was examined as a function of experimental condition (testing, restudy) and block (before study of list 3, after study of list 3).

In addition to frequentist analysis, Bayesian statistics were calculated in order to evaluate the degree of evidence in favor of null and alternative hypotheses. *BF*_01_ is reported when the Bayesian analysis provides relatively more evidence for the null hypothesis than for the alternative hypothesis; *BF*_10_ is reported when the analysis provides relatively more evidence for the alternative hypothesis than for null hypothesis (see Wagenmakers et al., 2018). To determine the strength of evidence, we used Jeffreys’s (1961) benchmarks, with Bayes factors corresponding to anecdotal (0–3), substantial (3–10), strong (10–30), very strong (30–100) or decisive (>100) evidence in favor of the null (*BF*_10_) or alternative hypothesis (*BF*_10_). All frequentist and Bayesian statistics were calculated with JASP (v 0.15; JASP Team, 2021). All data and analyses are stored in JASP files on OSF (*https://osf.io/v4cqu/*).

## Results

### Experiment 1a

#### List Learning Task

**Figure 2:**
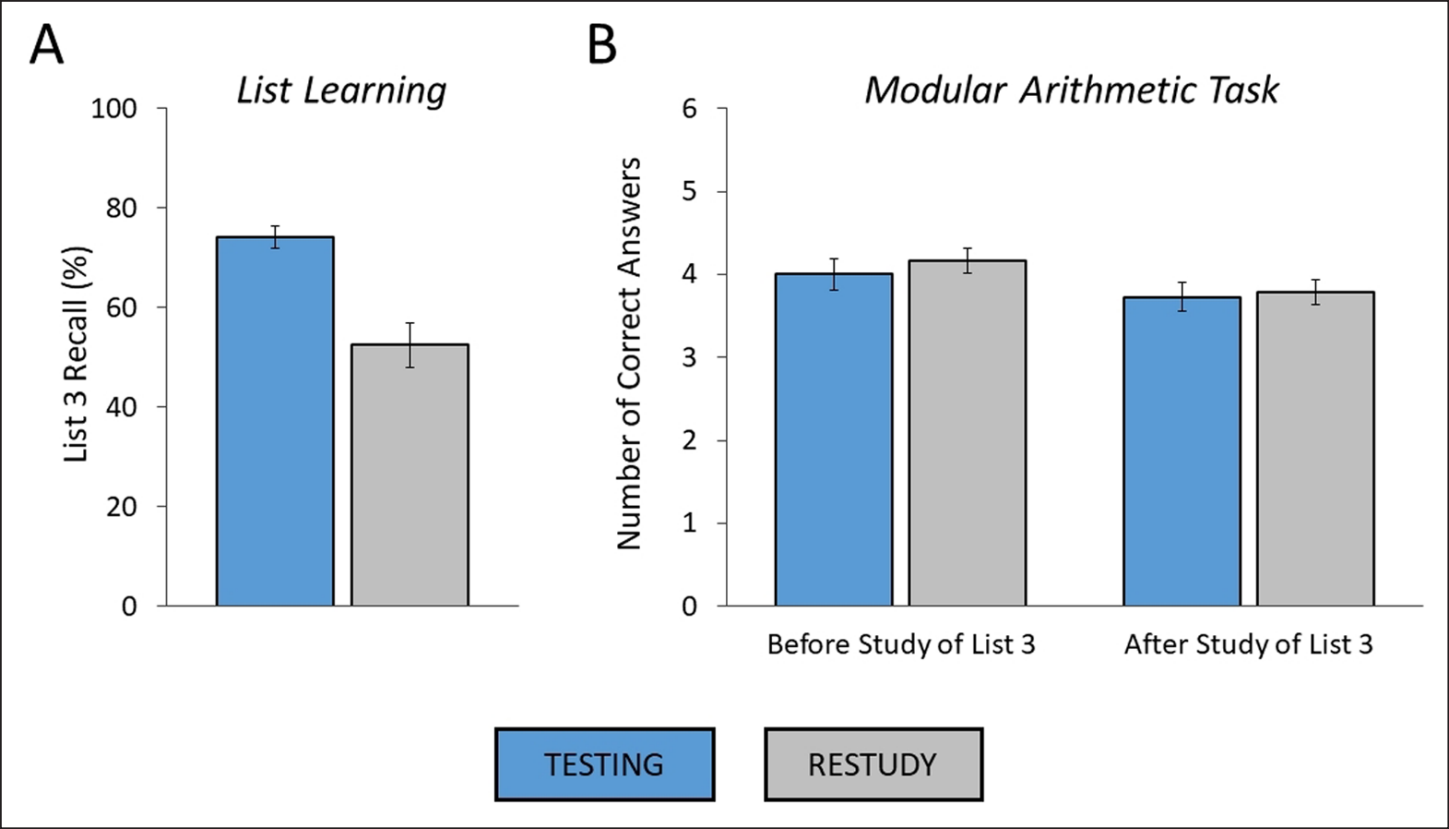
**(A, B)** Results of Experiment 1a. (A) List learning task. List 3 recall rates as a function of experimental condition (testing, restudy). (B) Modular arithmetic task. Number of correct answers as a function of block (before study of list 3, after study of list 3) and experimental condition (testing, restudy). Error bars: standard errors of the mean.

Descriptive statistics for all recall tests are shown in ***Table 1***. With regard to list 3 recall, a significant forward testing effect with higher correct recall of list 3 items in the testing condition than in the restudy condition was observed, *t*(63) = 6.90, *p* < .001, *d* = .862 (*BF*_10_ > 100, decisive evidence; one-tailed; see ***Figure 2A***). In addition, prior-list intrusions in the list 3 recall test were significantly reduced in the testing compared to the restudy condition, *t*(63) = –2.28, *p* = .026, *d* = –.285 (*BF*_10_ = 2.99, anecdotal evidence; one-tailed; see ***Table 1***).

**Table 1:** Results of the list learning task: Recall rates as a function of condition in Experiments 1a and 1b. Means and standard errors of the means (in parentheses).


				RECALL RATES				INTRUSIONS
		
EXPERIMENT	TEST	CONDITION		LIST 1	LIST 2	LIST 3		LIST 3

*Experiment 1a*	Immediate Recall	**Testing**		70.70 (2.19)	73.31 (2.14)	74.22 (2.19)		0.06 (0.03)

		**Restudy**				52.47 (3.52)		0.37 (0.13)

	Final Recall	**Testing**		43.23 (4.04)	49.74 (3.99)			

		**Restudy**		57.55 (3.64)	55.60 (3.80)			

*Experiment 1b*	Immediate Recall	**Testing**		65.23 (2.33)	66.54 (2.92)	65.23 (2.34)		0.25 (0.14)

		**Restudy**				49.74 (3.54)		0.44 (0.17)

	Final Recall	**Testing**		42.84 (3.75)	48.18 (3.75)			

		**Restudy**		59.90 (3.06)	50.13 (3.66)			


Regarding the immediate recall rates of lists 1 to 3 in the testing condition, an ANOVA with the factor of list (list 1, list 2, list 3) was calculated, which showed no significant effect of list, *F*(2,126) = 1.44, *MSE* = 147.63, *p* = .240 (*BF*_01_ = 5.39, substantial evidence, compared to null model; see ***Table 1***). Regarding the final recall rates of lists 1 and 2, no difference between the testing condition and the restudy condition was found for list 2, *t*(63) = –1.21, *p* = .231 (*BF*_01_ = 3.65, substantial evidence; two-tailed), whereas final list 1 recall was significantly reduced in the testing compared to the restudy condition, *t*(63) = –3.61, *p* < .001, *d* = –.452 (*BF*_10_ = 41.35, strong evidence; two-tailed; see ***Table 1***).

#### Modular Arithmetic Task

The results of the modular arithmetic task are shown in ***Table 2*** and depicted in ***Figure 2B***. The ANOVA for the number of correct answers with the factors of experimental condition (testing, restudy) and block (before study of list 3, after study of list 3) revealed a significant main effect of block, *F*(1,63) = 4.89, *MSE* = 317.72, *p* = .031, \eta _p^2 = .072 (*BF*_10_ = 1.77, anecdotal evidence; compared to null model), indicating a performance decrease from the first to the second block. More importantly, the analysis showed neither a significant main effect of condition, *F*(1,63) < 1 (*BF*_01_ = 5.78, substantial evidence; compared to null model), nor a significant interaction between the two factors, *F*(1,63) < 1 (*BF*_01_ = 5.09, substantial evidence; compared to two-main-effects model). Thus, these results suggest that participants’ performance in the modular arithmetic task was unaffected by preceding retrieval practice.

**Table 2:** Results of the arithmetic tasks (Exp. 1a: modular arithmetic problems, Exp. 1b: single-digit multiplication task). Number of correct answers as a function of condition and block. Means and standard errors of the means (in parentheses).


EXPERIMENT	BLOCK	CONDITION	CORRECT ANSWERS

*Experiment 1a*	Before Study of List 3	**Testing**	4.00 (0.19)

		**Restudy**	4.16 (0.15)

	After Study of List 3	**Testing**	3.73 (0.17)

		**Restudy**	3.78 (0.15)

*Experiment 1b*	Before Study of List 3	**Testing**	8.19 (0.47)

		**Restudy**	7.75 (0.53)

	After Study of List 3	**Testing**	8.84 (0.51)

		**Restudy**	8.89 (0.52)


### Experiment 1b

#### List Learning Task

**Figure 3:**
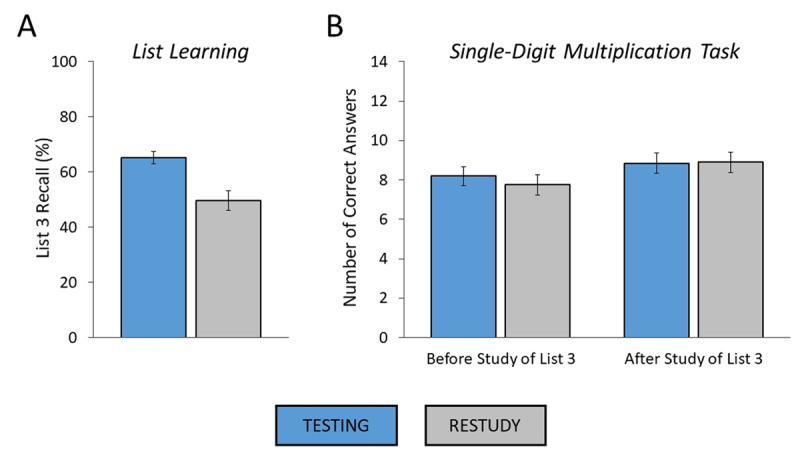
**(A, B)** Results of Experiment 1b. (A) List learning task. List 3 recall rates as a function of experimental condition (testing, restudy). (B) Single-digit multiplication task. Number of correct answers as a function of block (before study of list 3, after study of list 3) and experimental condition (testing, restudy). Error bars: standard errors of the mean.

Descriptive statistics for the recall tests are shown in ***Table 1***. The list 3 recall results revealed a significant forward testing effect with higher correct recall of list 3 items in the testing condition than in the restudy condition, *t*(63) = 5.22, *p* < .001, *d* = .652 (*BF*_10_ > 100, decisive evidence; one-tailed; see ***Figure 3A***). In contrast, no significant difference between conditions was observed regarding prior-list intrusions in the list 3 recall test, *t*(63) = –0.85, *p* = .199 (*BF*_01_ = 3.24, substantial evidence; one-tailed; see ***Table 1***).

Regarding immediate recall rates of the three lists in the testing condition, the ANOVA revealed no significant differences between lists, *F*(2,126) < 1 (*BF*_01_ = 16.68, strong evidence; see ***Table 1***). Regarding final recall rates of lists 1 and 2, no difference between the testing and restudy condition was observed for list 2, *t*(63) < 1 (*BF*_01_ = 6.59, substantial evidence; two-tailed), whereas final list 1 recall was significantly reduced in the testing condition compared to the restudy condition, *t*(63) = –4.83, *p* < .001, *d* = –.604 (*BF*_10_ > 100, decisive evidence; two-tailed; see ***Table 1***).

#### Single-Digit Multiplication Task

The results of the single-digit multiplication task are shown in ***Table 1*** and depicted in ***Figure 3B***. The ANOVA for number of correct answers with the factors of experimental condition (testing, restudy) and block (before study of list 3, after study of list 3) revealed a significant main effect of block, *F*(1,63) = 9.42, *MSE* = 5.49, *p* = .003, \eta _p^2 = .130 (*BF*_10_ = 6.72, substantial evidence; compared to null model), indicating a performance increase from the first to the second block. More importantly, the analysis showed neither a significant main effect of condition, *F*(1,63) < 1 (*BF*_01_ = 6.24, substantial evidence; compared to null model), nor a significant interaction between the two factors, *F*(1,63) < 1 (*BF*_01_ = 15.61, strong evidence; compared to two-main-effects model). Thus, participants’ performance in the single-digit multiplication task was unaffected by preceding retrieval practice.

### Joint Analysis of Experiments 1a and 1b

Because data collections of the two experiments were carried out during the same period of time and the 128 participants were randomly assigned to one of the two experiments, we ran a joint analysis of the data that were collected in the arithmetic tasks of Experiments 1a and 1b. The data were z-transformed with the means and standard deviations of the person factors, respectively (z-transformed data and statistics are stored as a JASP file on OSF; *https://osf.io/v4cqu/*).

We calculated an ANOVA for the z-transformed data with the factors of experimental condition (testing, restudy), block (before study of list 3, after study of list 3), and experiment (Experiment 1a, Experiment 1b). The analysis revealed a significant interaction between block and experiment, *F*(1,126) = 11.72, *MSE* = 1.01, *p* < .001, \eta _p^2 = .085 (*BF*_10_ = 44.22, very strong evidence; compared to three-main-effects model), which indicates that performance decreased from the first to the second block in Experiment 1a but increased in Experiment 1b. All other main effects and interactions were not significant, all *Fs*(1,126) < 1. Importantly, Bayesian analysis indicated substantial evidence in favor of the null hypothesis regarding both the main effect of condition (*BF*_01_ = 9.82, compared to null model) and the interaction between condition and experiment (*BF*_01_ = 4.84, compared to three-main-effects model), suggesting that, neither in Experiment 1a nor in Experiment 1b, there was an effect of retrieval practice on arithmetic task performance.

## Discussion

In both experiments, a reliable forward testing effect in list 3 recall rates was found, which replicates the findings from earlier studies on the forward testing effect (e.g., Bäuml & Kliegl, 2013; Szpunar et al., 2008; Pastötter et al., 2018; Pastötter & Frings, 2019). In addition, the results of Experiment 1a showed a significant reduction of prior-list intrusions in the list 3 recall test in the testing condition compared to the restudy condition. No such difference in prior-list intrusions was observed in Experiment 1b. However, because intrusions were produced very infrequently overall, the intrusion results should be interpreted with caution due to possible floor effects. More importantly, the results of both experiments provided substantial evidence against an influence of retrieval practice on participants’ performance in the subsequent arithmetic tasks, i.e., the modular arithmetic task in Experiment 1a and the single-digit multiplication task in Experiment 1b. No significant effect of testing on these tasks was observed either before or after list 3 learning.

Regarding the memory task, the list 3 recall results are consistent with both release-from-PI (Bäuml & Kliegl, 2013; Szpunar et al., 2008) and reset-of-encoding theories of the forward testing effect (Pastötter, Engel, & Frings, 2018; Pastötter et al., 2011). In addition, the finding of comparable list 1, list 2, and list 3 recall rates in the testing condition is consistent with both theories of the forward testing effect. Regarding the arithmetic tasks, however, only the release-from-PI theory is consistent with the present results. Indeed, build-up and release from PI should be specific to the memory task and switching to the arithmetic tasks should not result in any interference effects from preceding list learning and retrieval practice activities in these (working memory) tasks. In contrast, the reset-of-encoding theory is challenged by the present results. This theory assumes that testing reduces subsequent memory load and inattention and thus predicts a potentially positive effect of testing on participants’ performance in subsequent arithmetic tasks. However, no such positive effect was observed in the present results, neither in the first block of arithmetic problems before list 3 learning nor in the second block after list 3 learning. Although there has been a variety of supporting evidence for the reset-of-encoding theory from both behavioral studies (e.g., serial position analysis; Dang et al., in press; Pastötter et al., 2018; motor sequence learning; Tempel & Frings, 2019) and electrophysiological research (Pastötter et al., 2011), other recent findings seem to challenge this theory (e.g., mediation analysis; Yang et al., in press). Thus, regarding the reset-of-encoding theory, further theoretical exploration is required in future research.

The results of Experiment 1a challenge the cognitive load theory (Chen et al., 2018; Leahy & Sweller, 2019), according to which the participants’ working memory resources should have been depleted after retrieval practice and therefore performance in the modular arithmetic task should have been impaired after testing compared to restudy. In addition, cognitive load theory is challenged by the results of the joint analysis, which provides substantial evidence against an interaction between the factors of condition and experiment. Indeed, if we assume that participants’ working memory load was relatively high in the more demanding modular arithmetic task in Experiment 1a but relatively low in the single-digit multiplication task in Experiment 1b, cognitive load theory predicts an ordinal interaction between condition and experiment, due to relatively larger working memory resource depletion after retrieval practice in Experiment 1a than in Experiment 1b. However, this is not what the results of the joint analysis showed. Notably, Leahy and Sweller (2019) argued that element interactivity of the studied material needs to be high in order to observe a detrimental effect of retrieval practice on subsequent working memory performance. According to this view, an effect may have been missed in the present study because unrelated word lists have low element interactivity. However, we think that the argumentation of Leahy and Sweller (2019) was based on inconclusive evidence. In their first experiment, in which element interactivity was considered low, retrieval practice did not affect working memory performance, neither after short nor after long delay. In their second experiment, in which element interactivity was considered high, the interaction between retrieval practice and restudy conditions and delay was not significant and therefore also the second experiment failed to provide clear evidence for an effect of retrieval practice on subsequent working memory performance. Thus, future studies using the present multi-list learning environment with more complex material are needed to investigate to what extent element interactivity may influence the results.

The forward testing effect is typically studied in a multi-list learning environment where participants study and retrieve items from the same type of material (e.g., words). Yang et al. (2019) have recently shown that the forward testing effect can be reliably observed even when material types are switched from list to list, or from block to block. For example, in one experiment, participants studied object pictures in the first block, prose in the second block, and face-profession pairs in the third block. Participants either restudied or were tested on the pictures (with a recognition test) in the first bock and the prose (with a fill-in-blank test) in the second block. All participants were tested on the face-profession pairs in a cued recall test in the third block. The results of this cued recall test showed a significant benefit of forward testing, which suggests that the forward testing effect is transferable even when material types and test formats are switched from list to list, or from block to block. Because the findings are difficult to be explained by release-of-PI and reset-of-encoding theories, Yang et al. (2019) suggested a combined test-expectancy and retrieval-effort theory to account for the transfer of the forward testing effect to the different materials or domains. In the present study, no benefits of retrieval practice on subsequent performance in the arithmetic (working memory) tasks was observed. Taken together, then, these findings suggest that the forward testing effect transfers to different materials or domains within episodic memory (tasks) but does not transfer from episodic memory to working memory (tasks).

The present study suggests that retrieval practice does not (causally) influence participants’ subsequent working memory performance. In addition, earlier studies, which followed the individual-differences approach, demonstrated that both direct and indirect benefits of testing are unrelated to individuals’ working memory capacity, as measured with complex working memory tasks such as the operation or symmetry span tasks. This holds for the backward testing effect (Agarwal, Finley, Rose, & Roediger, 2017; Aslan & Bäuml, 2011), the forward testing effect (Pastötter & Frings, 2019; Yang et al., 2020), and test-potentiated learning (Bertilsson, Wiklund-Hörnqvist, Stenlund, & Jonsson, 2017; Brewer & Unsworth, 2012). Arguably, all these studies examined individual differences in working memory performance and the benefits of retrieval practice in healthy younger adults. Therefore, it needs to be shown whether these findings generalize to other populations, i.e., children, older adults, and patient groups. Based on the present study and the earlier individual-differences research, it can be concluded that the effectiveness of retrieval-based learning does not depend to a significant degree on adults’ working memory capacity, nor does retrieval practice in the present multi-list learning environment affect adults’ performance in subsequent working memory (i.e., arithmetic) tasks.

Regarding the final recall of lists 1 and 2, the results of the two experiments revealed benefits of restudy over retrieval practice for list 1 but no difference between conditions for list 2. Actually, this is an expected finding, which has also been observed in earlier research (e.g., Pastötter & Frings, 2019). First, the difference between conditions in the final list 1 recall rates can be considered as a rough measure of the backward testing effect (note that there was retroactive interference and also output interference from lists 2 and 3 during recall testing of list 1). Thus, the finding that restudied list 1 items were better recalled than previously tested list 1 items is consistent with the literature, showing that the backward testing effect is most prominent when the final recall testing is administered after a relatively long delay (e.g., 2 days) but is often eliminated or even reversed when final recall testing is administered after a relatively short delay (e.g., 5 min; see Roediger & Karpicke, 2006; Toppino & Cohen, 2009). Second, the difference between conditions in the final list 2 recall rates provided a mixed measure of backward and forward effects (with additional retroactive interference and output interference from list 3), which can explain why no significant difference between conditions was observed.

To conclude, the results of two experiments suggest that retrieval practice in a multi-list learning environment does not influence adult participants’ performance in subsequent arithmetic tasks, which rely on individual working memory resources. Together with the findings from previous research on benefits of retrieval practice for long-term memory and learning, the present study suggests that retrieval practice is an effective learning technique that comes without indirect costs for other unrelated (working memory) tasks.

## Data Accessibility Statements

Material and data can be found at Open Science Framework, *https://osf.io/v4cqu/*, DOI: *10.17605/OSF.IO/V4CQU*.

## Footnotes

It should be noted that testing previously studied information can sometimes have detrimental effects on subsequent new learning. For example, in the memory updating paradigm, testing has been shown to impair subsequent encoding on a trial-by-trial basis compared to relearning (Davis & Chan, 2015; Finn & Roediger, 2013). However, there are a number of procedural differences between this memory updating paradigm and the present multi-list paradigm (e.g., testing with immediate feedback on a trial-by-trial basis and self-paced test trials in the memory updating paradigm), which may explain the different results.
